# The complete mitochondrial genome of *Rhynchocinetes brucei* Okuno 1994 (Decapoda: Rhynchocinetidae)

**DOI:** 10.1080/23802359.2023.2261636

**Published:** 2024-03-11

**Authors:** Hangjun Wang, Sheng Zeng, Zhao Zhang, Deyuan Yang

**Affiliations:** a Wenzhou Marine Center, Ministry of Natural Resources of the People’s Republic of China, Beijing, China; b Marine Ecosystem Observation and Research Station on the Yangtze River Estuary, Wenzhou, China; c College of the Environment and Ecology, Xiamen University, Xiamen, China; d National Taiwan Ocean University, Keelung, Taiwan, China

**Keywords:** *Rhynchocinetes*, East China Sea, mitogenome, phylogenetic analysis

## Abstract

We report the complete mitochondrial genome of *Rhynchocinetes brucei* Okuno 1994. The mitogenome was found to contain 16158 bp with 13 protein-coding genes (PCGs), 22 tRNA genes (tRNAs), 2 rRNA genes (rRNAs), and 1 putative control region. Phylogenetic analysis indicated that *R. brucei* was sister to *Rhynchocinetes durbanensis* (PP= 1), of the same family Rhynchocinetidae. These results are helpful for research on the phylogenetic and evolutionary studies of this group.

## Introduction

**Figure 1.**
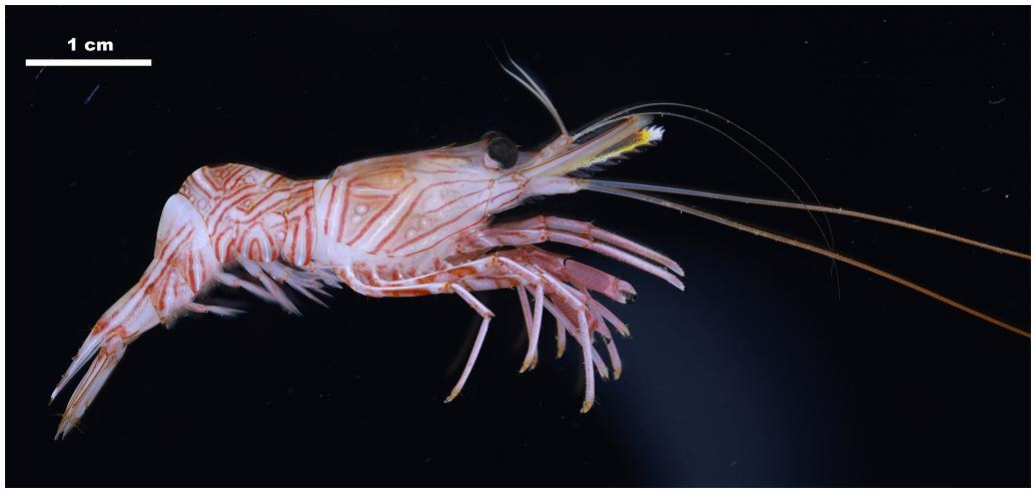
The studied specimen of *R. brucei* (voucher no.: MBM189207). Photograph by Hangjun Wang.

*Rhynchocinetes brucei* (Decapoda: Rhynchocinetidae) is widely distributed in the Philippines, Hong Kong, and the Great Barrier Reef in the tropical West Pacific (Okuno 1994). The mate guarding behavior displayed by *R. brucei* is rarely observed among caridean shrimp (Martin et al. 2010). The distinguishing features of this species include intricate red bands that cover the entire surface, a notable dark red spot located on the back of the third abdominal segment (Figure 1), and articular gills on its first three pairs of pereiopods (Okuno 1994). There is only one complete mitochondrial genome of the family Rhynchocinetidae in the NCBI GenBank (*Rhynchocinetes durbanensis*, KT590405). In this study, we first recorded this species in the East China Sea and reported the complete mitochondrial genome of *R. brucei*, which provided molecular information for research on the phylogenetic and evolutionary studies of this group.

## Materials

The studied specimen of *R. brucei* used in this study was collected from rocks of Nanji Islands, East China Sea (27°27'28.24"N, 121°4'39.63"E, water depth 6 m) on 18 July 2021. The specimen was fixed and preserved in 100% ethanol, and finally deposited in the Institute of Oceanology, Chinese Academy of Sciences (IOCAS) in Qingdao, China (contact: Hangjun Wang, wanghj@ecs.mnr.gov.cn) under the voucher number MBM189207.

## Methods

DNA extraction, sequencing, and clean data were performed according to the methods used by Li et al. (2023). The raw data, including 34,590,902 reads amounting to 5.19 G. To check the consistency of results from different assembly strategies, two methods, i.e. GetOrganelle v1.7.6.1 (Jin et al. 2020) and NovoPlasty v. 4.3.1 (Dierckxsens et al. 2017) were used for assembly.

To evaluate the quality of assembly, clean reads were mapped to the obtained contig using a subcommand mitoz visualize of MitoZ v3.6 (Meng et al. 2019) to show the coverage depth. The coverage depth of the assembled mitochondrial genome was calculated by applying the samtools depth command in Samtools v.1.6 (Danecek et al. 2021), and a coverage map was generated using Circos v.0.69 (Krzywinski et al. 2009). Annotation of the mitochondrial genome was carried out using the MITOS2 (Donath et al. 2019) and the MitoZ annotation module (Meng et al. 2019). The two results of annotation were loaded into Geneious v2021.0.3. and checked manually with the help of ORFs (finds open reading frames). The final mitogenome sequence was visualized using the visualize subcommand in MitoZ v3.6 (Meng et al. 2019). Strand asymmetry was calculated using the formulae: AT-skew = (A − T)/(A + T); GC-skew = (G − C)/(G + C) (Perna and Kocher 1995).

We selected the newly sequenced mitogenome of *R. brucei* and the complete mitogenomes of 18 other shrimp species from 8 families: Alpheidae Rafinesque, 1815; Palaemonidae Rafinesque, 1815; Crangonidae Haworth, 1825; Lysmatidae Dana, 1852; Hippolytidae Spence Bate, 1888; Rhynchocinetidae Ortmann, 1890; Thoridae Kingsley, 1878; and Pandalidae Haworth, 1825. For each genus in these families, one sequence was included (Table 1). We performed phylogenetic analyses of this dataset using PhyloSuite 1.2.3 (Zhang et al. 2020).

**Table 1.** Mitochondrial sequences used in the phylogenetic analysis as shown in Figure 3.

Family	Species name	Genbank accession no.	Length (bp)	References
Alpheidae	*Leptalpheus forceps*	MN732884	15463	Scioli et al. 2020
Alpheidae	*Alpheus hoplocheles*	MG873459	15735	Zhong et al. 2019
Palaemonidae	*Macrobrachium bullatum*	KM978918	15774	Gan et al. 2016
Palaemonidae	*Palaemon serenus*	KM978916	15967	Gan et al. 2016
Palaemonidae	*Hymenocera picta*	MF804409	15786	Sung et al. 2018
Palaemonidae	*Anchistus australis*	MN412556	15396	Liu 2020
Pandalidae	*Pandalus hypsinotus*	MH920259	15909	Kim et al. 2019
Pandalidae	*Heterocarpus ensifer*	MF804409	15939	Sun et al. 2020
Pandalidae	*Chlorotocus crassicornis*	KY944589	15935	Kim et al. 2019
Pandalidae	*Plesionika edwardsii*	OP087601	15956	Jimenez-Ruiz et al. 2022
Pandalidae	*Bitias brevis*	MG674229	15891	Sun et al. 2020
Crangonidae	*Crangon hakodatei*	KU641481	16060	Kim and Kim 2016
Lysmatidae	*Lysmata boggessi*	MK932871	17345	Genbank
Lysmatidae	*Exhippolysmata ensirostris*	MK681888	16350	Ye et al. 2021
Thoridae	*Thor amboinensis*	MT671809	15553	Wang et al. 2020
Thoridae	*Lebbeus groenlandicus*	MN577077	17399	Kim et al. 2019
Hippolytidae	*Saron marmoratus*	MT795210	16330	Wang et al. 2021
Rhynchocinetidae	*Rhynchocinetes durbanensis*	KT590405	17695	Tang et al. 2016
**Rhynchocinetidae**	***Rhynchocinetes brucei***	**OR095174**	**16158**	**This study**

Each coding gene was aligned individually using Mafft v.7.313 (Katoh and Standley 2013), with codon alignment mode applied. Ambiguously aligned regions were removed using Gblocks v.0.91 with default settings (Castresana 2000). The best substitution models of each partition (genes) were selected with ModelFinder v2.2.0 (Kalyaanamoorthy et al. 2017).

Bayesian Inference phylogenies were inferred using MrBayes v3.2.7a (Ronquist et al. 2012) under a partition model (2 parallel runs, 2000000 generations), in which the initial 25% of sampled data were discarded as burn-in. Finally, we used iTOL v6 (Letunic and Bork 2016) to visualize the derived BI tree.

## Results

**Figure 2.**
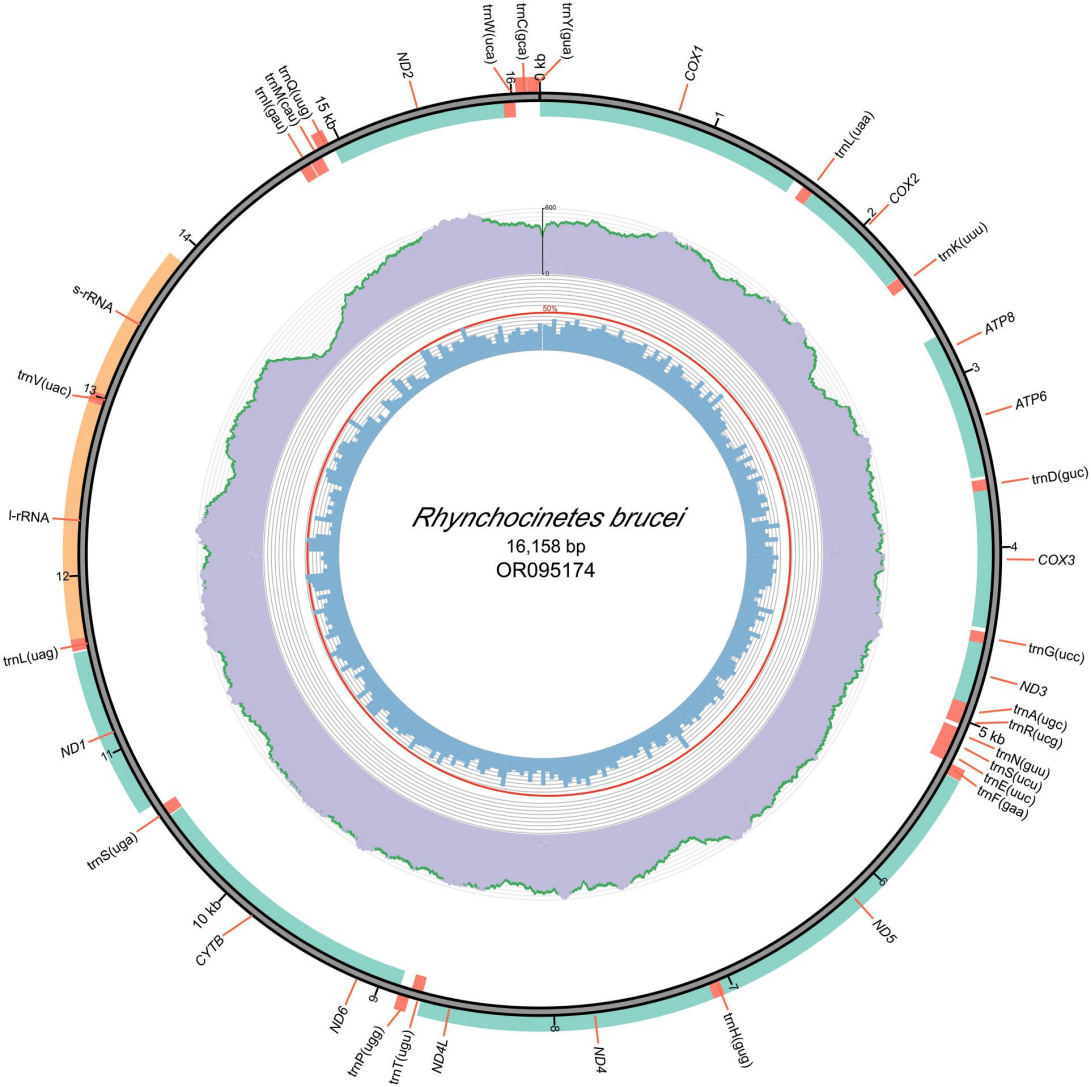
The complete mitogenome of *R. brucei*. The innermost and Middle circles depict the GC content and distribution of sequencing depth, respectively. The outermost circle indicates the arrangements of genes: inner genes from the forward strand, and outer genes from the reverse strand, with PCGs in green, rRNAs in orange, and tRNAs in red.

The results obtained from both the GetOrganelle and NovoPlasty approaches for assembling the mitogenome demonstrated consistency (16158 bp) and their average coverage of 450 and 442, respectively. The total length of the mitochondrial genome was 16,158 bp (GenBank accession number: OR095174), with a high A + T content of 71.7% (38.6% T and 33.1% A), and lower levels of C and G at 17.5% and 10.8%, respectively. Both AT-skew and GC-skew of the mitogenome are negative, −0.076 and −0.239, respectively. Our analysis revealed that the genome contained 13 protein-coding genes (PCGs), 2 ribosomal RNA (rRNA) genes, 22 transfer RNA (tRNA) genes, and 1 putative control region measuring 848 bp (Figure 2). Five PCGs (*COX2*, *ATP8*, *ATP6*, *ND4L*, and *CYTB*) started with the ATG codon, three (*COX3*, *ND6*, and *ND1*) started with ATA, *ND5* and *ND2* started with ATT, *COX1* started with CGA, *ND3* started with ATC, *ND4* started with GTG. As for the stop codons, seven PCGs (*COX1*, *COX2*, *ATP8*, *COX3*, *ND4L*, *CYTB*, and *ND2*) ended with TAA, four (*ATP6*, *ND3*, *ND4*, and *ND1*) with TAG and two (*ND5* and *ND6*) with T.

**Figure 3.**
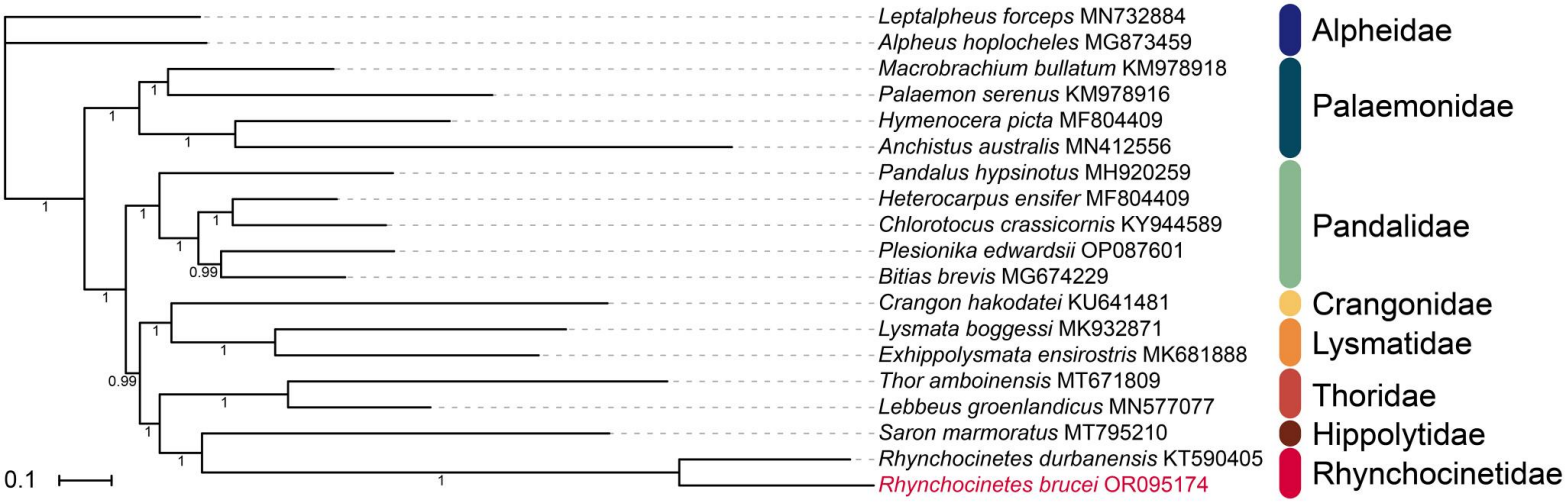
Phylogenetic analysis (Bayesian Inference) of *R. brucei* on the whole mitochondrial genome sequences. The tree was constructed based on concatenated nucleotide sequences of 13 protein-coding genes (PCGs) of 19 species in 8 families. The numbers under the internodes represent Bayesian inference (BI) posterior probabilities (PP). GenBank accession numbers used are listed after the species names. The scale bar indicates the number of substitutions per site.

The BI phylogenetic tree, based on 13 PCGs, showed that *R. brucei* was the sister to *R. durbanensis* (PP = 1), and Rhynchocinetidae was closer to Hippolytidae (Figure 3).

## Discussion and conclusion

In this study, we first reported the full mitochondrial genome of *R. brucei* and gave a detailed annotation. To ensure the reliability of the assembly, we employed two distinct assembly methods, namely GetOrganelle and NovoPlasty. We found that *ATP8* gene of the *R. brucei* is difficult to annotate using MITOS2 and MitoZ, so we annotated it using find ORFs function in Geneious and compared it with other mitochondrial genomes in the infraorder Caridea. Other technologies such as transcriptomics sequencing may be helpful for the reliability of *ATP8* annotation.

## Data Availability

The genome sequence data that support the findings of this study are openly available in GenBank of NCBI under accession no. OR095174. The associated BioProject, Bio-Sample, and SRA numbers are PRJNA984426, SAMN35767296, and SRR24962457, respectively.
